# Retinoblastoma Tumor Suppressor Protein Roles in Epigenetic Regulation

**DOI:** 10.3390/cancers12102807

**Published:** 2020-09-29

**Authors:** Frederick Guzman, Yasamin Fazeli, Meagan Khuu, Kelsey Salcido, Sarah Singh, Claudia A. Benavente

**Affiliations:** 1 Department of Pharmaceutical Sciences, University of California, 101 Theory #100, Irvine, CA 92617, USA; guzmanfg@uci.edu; 2 School of Biological Sciences, University of California, 5120 Natural Sciences II, Irvine, CA 92697, USA; yfazeli@uci.edu (Y.F.); mskhuu@uci.edu (M.K.); salcidok@uci.edu (K.S.); singhss1@uci.edu (S.S.); 3 Department of Developmental and Cell Biology, University of California, 2011 Biological Sciences III, Irvine, CA 92697, USA; 4 Chao Family Comprehensive Cancer Center, University of California, 839 Medical Sciences Rd, Irvine, CA 92697, USA

**Keywords:** retinoblastoma, RB1, E2F, epigenetic, DNA methylation, histone modification, nucleosomes, miRNA, ncRNA, chromatin

## Abstract

**Simple Summary:**

Loss of function of the retinoblastoma gene (*RB1*) is the rate-limiting step in the initiation of both the hereditary and sporadic forms of retinoblastoma tumor. Furthermore, loss of function of the retinoblastoma tumor suppressor protein (pRB) is frequently found in most human cancers. In retinoblastoma, tumor progression is driven by epigenetic changes following pRB loss. This review focuses on the diverse functions of pRB in epigenetic regulation.

**Abstract:**

Mutations that result in the loss of function of pRB were first identified in retinoblastoma and since then have been associated with the propagation of various forms of cancer. pRB is best known for its key role as a transcriptional regulator during cell cycle exit. Beyond the ability of pRB to regulate transcription of cell cycle progression genes, pRB can remodel chromatin to exert several of its other biological roles. In this review, we discuss the diverse functions of pRB in epigenetic regulation including nucleosome mobilization, histone modifications, DNA methylation and non-coding RNAs.

## 1. Introduction

**Figure 1:**
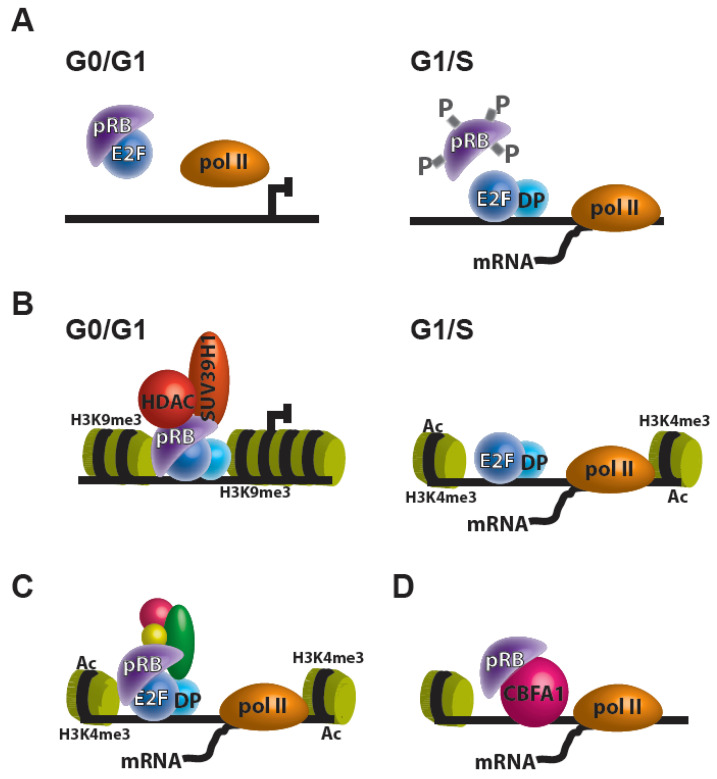
Transcriptional regulation by pRB. (**A**–**C**) The pRB can regulate E2F targets in at least three different mechanisms: (**A**) direct pRB repression on E2F transcription; (**B**) pRB recruitment of transcriptional corepressors, like HDACs and histone methyltransferases (e.g., SUV39H1) to E2F targets; and (**C**) association of the pRB-E2F complex with transcriptional coactivators, which results in increased expression of E2F targets. (**D**) pRB can regulate transcription through its interaction with other transcription factors (e.g., CBFA1).

**Figure 2:**
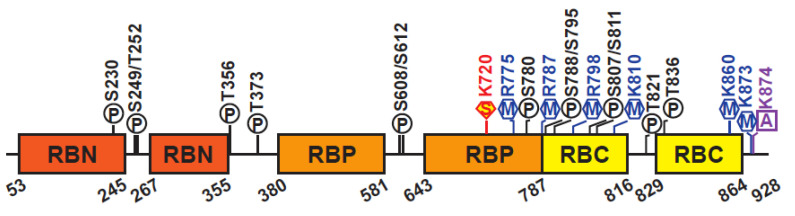
Schematic of pRB structure. Representation of the human pRB domain organization and post-translational modifications. Structured domains are depicted as colored boxes, unstructured domains are depicted as a line in black. Phosphorylation (P) sites are shown in black, methylation (M) sites in blue, acetylation (A) sites in purple, and sumoylation (S) sites in red.

Mutations leading to the loss of the retinoblastoma transcriptional corepressor 1 gene (*RB1*) or deregulation of its gene product, the retinoblastoma tumor suppressor protein (pRB), are associated with several forms of cancer including retinoblastoma, osteosarcoma, adenocarcinomas, small cell lung cancer, breast cancer, prostate cancer, and others [1]. pRB controls transcription and is a negative regulator of cell proliferation through repression of the E2F family of transcription factors (E2Fs). Transcriptional repression in this model occurs through the binding of pRB’s “pocket” domain to E2Fs’ C-terminal transactivation domain [2]. In addition to its interactions with E2Fs, pRB can further regulate transcription through the binding to chromatin remodelers. Current models of pRB functions are increasingly complex and yet to be completely elucidated. The pRB-E2F association is known to play a role in transcriptional regulation of E2F targets in at least three different mechanism: (1) direct pRB repression on E2F transcription (Figure 1A), (2) pRB recruitment of transcriptional corepressors to E2F targets (Figure 1B), and (3) association of the pRB-E2F complex with transcriptional coactivators, which result in increased expression of E2F targets (Figure 1C) [3]. It is also noteworthy that beyond its E2F transcriptional regulation, pRB may regulate transcription through its interaction with other transcription factors and has transcription-independent roles throughout the cell (Figure 1D) [4].

Within the cell cycle, CYCLIN D: CDK4/6 mono-phosphorylates pRB at active sites and unstructured regions of the protein during early G1 phase, CYCLIN E: CDK2 hyper-phosphorylates pRB in late G1, and CYCLIN B: CDK1 maintain pRB in a hyper-phosphorylated state during S, G2, and M phases [5]. pRB mono-phosphorylation inactivates unphosphorylated pRB G0 functions. pRB hyper-phosphorylation inhibits its ability to bind to E2Fs, allowing E2Fs to drive transcription of genes necessary for G1/S phase cell cycle progression. While hyper-phosphorylation largely disrupts pRB binding to other proteins, some protein interactions persist following pRB hyper-phosphorylation. The protein–protein interactions that co-exist with pRB in a hyper-phosphorylated form suggest that pRB hyper-phosphorylation does not inhibit all pRB functions [6]. While pRB binding to E2Fs occur at the pocket domain, pRB interactions with epigenetically-relevant proteins involve the LxCxE-binding domain [7]. The availability of the LxCxE binding site, even when pRB is bound to E2F, presents expanded functions of pRB in the recruitment of chromatin remodelers and other protein complexes. Indeed, pRB has been reported to interact with over 300 proteins and many protein interactions are, or are involved with, chromatin modifier proteins [8]. pRB therefore extends relevance into protein complexes beyond those within the canonical pRB/E2F pathway. Studies in retinoblastoma indicate that these tumors develop quickly as a result of epigenetic deregulation of key cancer pathways as a direct result of pRB loss [9]. Studies in the last decade have found significant evidence regarding the relationship between pRB and chromatin remodeling proteins, pushing the boundaries of knowledge regarding pRB functions. This review provides an in-depth examination of the literature involving pRB in epigenetic regulation, including nucleosome mobilization, histone acetylation, histone methylation, DNA methylation, and non-coding RNAs.

## 2. pRB Protein Structure

Human pRB consists of 928 amino acids, which can be divided into three domains: the N-terminal domain (RBN), the A/B region or “pocket” domain (RBP), and the C-terminal domain (RBC) (Figure 2) [10,11]. The RBP contains a Leu-X-Cys-X-Glu (LxCxE) binding cleft which mediates interactions with hundreds of proteins, including oncoproteins and transcription factors. Among these, the best characterized interaction is that between pRB and the E2F family of transcription factors. The interaction between pRB and other proteins depends on the pRB’s structure and its post-translational modifications, which play a central role in regulating pRB’s function in many different developmental processes (reviewed in [8,12,13]). The RBN domain closely resembles the pocket structure and can physically interact with the RBP [14]. The RBC domain is intrinsically disordered and is required for the interaction between pRB and the E2F/DP complex [15].

The three pRB domains are connected by unstructured linker sequences that provide flexibility to the protein. These linker sequences are subjected to post-translational modifications, most notably CDK-dependent phosphorylation, affecting pRB interaction with other proteins and its functional roles [16,17]. In general, pRB phosphorylation results in inactivation of pRB functions, resulting in transcriptional derepression and cell cycle progression [18]. Human pRB has 14 phosphorylation, 2 acetylation, and 6 methylation sites, and is also subject to ubiquitination and SUMOylation (Figure 2) [8,13]. These post-translational modifications can promote or prevent occurrence of other modifications, controlling pRB functions, including its ability to recruit chromatin remodeling factors.

## 3. Role of pRB in Nucleosome Mobilization

Genomic DNA is packaged by histones and non-histone proteins into nucleosomes, the structural units of the eukaryotic chromatin and chromosomes. SWI/SNF complexes are multiprotein chromatin remodeling complexes found in eukaryotes that rearrange nucleosomes in an ATP-dependent manner [19,20,21,22]. These modifications can involve changing the location and/or changing the conformation of nucleosomes, thus reorganizing chromatin structure in order to facilitate transcription factor binding [23,24,25,26,27,28]. The nucleosomal changes mediated by SWI/SNF can result in transcriptional activation or repression, depending on the components of the complex. In mammals, SWI/SNF complexes are driven by the ATPase subunits Brahma (BRM) and Brahma-related gene 1 (BRG1) [29,30]. Both BRM and BRG1 contain a LxCxE binding motif and are known to interact with pRB [31,32,33,34].

BRG1 and BRM binding to pRB is crucial for pRB-mediated repression of E2F1 activity and the resulting cell cycle arrest [31,34]. BRM and BRG1-deficient cells are resistant to pRB-mediated arrest, an effect that is reversible through ectopic expression of BRM and BRG1 to re-sensitize cells to growth inhibition [35,36,37,38,39]. As BRM and BRG1 share some functional redundancy, BRM expression is sufficient for sensitizing cells to pRB-mediated arrest when BRG1 is lost [39]. It is therefore an established consensus that physical interaction between pRB and BRM/BRG1 is required for pRB-mediated cell cycle arrest and that the SWI/SNF complex is directly involved in cell cycle regulation. However, a study showing that cellular growth and senescence could still be achieved when the LxCxE motif is mutated in BRG1, brought into question whether the direct interaction between BRG1 and pRB is essential [40]. Although this finding could be disputed by an earlier study indicating that pRB and BRG1 do not interact through the LxCxE motif but through an adjacent site [41], the former study suggests that the induction of pRB dephosphorylation needed for cell cycle arrest is the result of BRG1-facilitated upregulation of the cyclin-dependent kinase inhibitor, p21 [40].

Despite being predominantly associated with transcriptional activation, BRG1 forms pRB-SWI/SNF and pRB-SWI/SNF-HDAC repressor complexes, acting as cell cycle checkpoints during G1-S transition by inhibiting CYCLIN A and CYCLIN E, respectively [31,33,34]. The formation and activity of these repressors are regulated by CDK-mediated phosphorylation of pRB [41] and BRM/BRG1 [42,43]. In addition to transcriptional regulation, pRB and BRG1 also interact during DNA double strand break repair [44]. Upon DNA damage, a phosphorylated E2F1-TopBP1 complex forms to recruit pRB. Then, pRB subsequently recruits BRG1 to the double strand break in order to decrease nucleosome density at the site of the break and stimulate DNA end resection [44]. In this complex, pRB also plays a stabilizing role by shielding phosphorylated E2F1 from proteasomal degradation [45].

It is incontestable that the interplay between pRB and SWI/SNF is critical for cellular homeostasis. SWI/SNF complexes are considered tumor suppressors and are mutated in nearly 20% of human malignancies [46]. Specifically, BRG1 is reported to be a target for mutation in 10–30% of human cancer cell lines [38,47,48]. In pRB wild-type cancers, harboring loss of or mutated BRG1 could contribute to pRB signaling pathway inactivation and thus provide growth advantage [36,37]. While loss of BRM/BRG1 is associated with clinical poor prognosis in non-small cell lung cancers [48], many others have suggested that elevated BRM/BRG1 levels is a prognostic factor for various types of cancers [49,50,51]. In addition, loss of *Brg1* in combination with pRB-loss enhances retinoblastoma tumorigenesis, suggesting that BRG1 acts as a tumor suppressor in the developing retina [52]. In summary, mammalian SWI/SNF complexes and their individual subunits BRM and BRG1 are closely associated with cellular processes regulated by pRB; however, the activity and mechanism of actions of these complexes remain highly context-dependent.

## 4. pRB and Histone Acetylation

Histone acetylation involves the transfer of an acetyl group from acetyl coenzyme A to lysine residues on histone tails by histone acetyltransferases (HATs). Histone acetylation relaxes the chromatin structure, allowing access of transcriptional activators to gene promoter regions to stimulate transcription. Several HATs have been identified, including transcriptional coactivators CBP/p300, PCAF, GCN5L, SRC1, ACTR and TAFII250. Removal of these acetyl groups from the tails of histone octamers is mediated by any of the 18 histone deacetylases (HDACs 1 to 18), ultimately influencing heterochromatinization of the nucleosome [53]. These modifications of the acetylation state of histone tails can be facilitated by the interaction of pRB protein with class I HDACs (HDAC1-3) within its role in E2F transcriptional repression during cell cycle regulation and terminal differentiation [54,55]. Furthermore, pRB also forms part of HAT complexes to facilitate HAT inactivation to repress transcription during terminal differentiation [56,57] or to facilitate histone acetylation required for DNA break repair [58].

The binding to HDACs and other chromatin remodelers is facilitated by the LxCxE motif within the pocket structure of pRB [7]. Since binding of E2F and pRB is independent of the LxCxE domain, HDAC1 and HDAC2 can interact with the pRB/E2F complex to help in the long-term repression of E2F target genes [54,59]. The interaction between pRB and HDAC1 can also be indirect: HDAC1-3 can bind to pRB using pRB-binding proteins, like RBP1, as bridging molecules [60] and HDAC1 is part of the SIN3 and CTBP/CTIP complexes, which are also pRB-interacting proteins [61,62]. The deacetylation activity of HDACs is required for pRB repression of genes normally activated during G1 [41]. Inhibition of HDAC activity, using inhibitors like Trichostatin A, was shown to inhibit the pRB-mediated inactivation of G1 cell cycle genes [41,63]. HDACs deacetylase activity when interacting with pRB is also important for the placement of further repressive chromatin modifications, as histone deacetylation is required prior to pRB’s association with SUV39H1 and HP1 for H3K9 trimethylation, discussed in the next section [64,65]. Beyond gene expression repression, the interactions between HDAC1/2 and the pRB/E2F complex mediate epigenetic silencing of transposable elements including long interspersed nuclear elements (LINE); thus, helping maintain genomic stability [66].

As mentioned previously, E2F1 and pRB also participate in transcription-independent functions in DNA repair [44,67]. In this process, interactions between HATs and pRB are an important component of the DNA damage response. Following the E2F1 and pRB recruitment of BRG1-containing SWI/SNF complex to double-strand breaks to decrease nucleosome density at the site of the DNA breaks [44], E2F1 and pRB recruit p300/CBP to sites of DNA damage [58]. p300 and CBP then mediate the acetylation of multiple lysine residues on histone H3 in nucleosomes flanking double-strand breaks. Further, E2F1 and pRB are also required for the recruitment of Tip60 and induction of H4K16 acetylation at DNA breaks, although this may involve an indirect mechanism rather than direct interaction between Tip60 and pRB [58].

## 5. pRB and Histone Methylation

Another important mechanism for chromatin regulation involves histone methylation. Histone methylation is catalyzed by histone methyltransferases (HMTs) and removal of these methyl groups is mediated by histone demethylases (KDMs). In humans, there are over 100 predicted HMTs and more than 20 KDMs. Histone methylation comprises an elaborate network that can activate or repress chromatin depending on the amino acid residue modified and the proteins that bind to these modified tails. Among the most studied histone methylation marks associated with repressed chromatin are histone H3 lysine 9 di- and tri-methylation (H3K9me2/3), histone H4 lysine 20 trimethylation (H4K20me3), and histone H3 lysine 27 trimethylation (H3K27me3). Several studies indicate that pRB can interact with HMTs and KDMs independent of the LxCxE motif to repress transcription as well as to maintain heterochromatin in intergenic zones, centromeres, peri-centromeres, and telomeres [64,68,69,70].

SUV39H1 is an HMT that functionally interacts with the heterochromatin protein 1 (HP1) to repress transcription at heterochromatic and euchromatic sites. SUV39H1 trimethylates H3K9, creating a repressive histone mark that serves as a binding site for HP1. The interaction between H3K9me3 and HP1 changes the nucleosome structure into a packed, transcriptionally inactive conformation. pRB can associate with SUV39H1 and HP1 through the pocket domain to repress transcription, including cell cycle genes cyclin E and cyclin A2 [64,69]. In this process, pRB is both necessary for the direct methylation of H3K9 by SUV39H1 and the binding of HP1 to repress gene expression [64]. The interaction between pRB and HP1, and the resulting changes in heterochromatin organization, also contribute to the changes in gene expression associated with the induction of senescence and the permanence of the senescent state [71].

pRB also binds to HMTs SUV4-20H1 and SUV4-20H2, two H4K20 trimethylating enzymes [70]. Trimethylation of H4K20 by SUV4-20H marks pericentric heterochromatin, a fraction of the heterochromatin located on both sides of centromeres that is crucial for preserving the integrity of the genome and promoting correct chromosomal segregation. The formation of this constitutive heterochromatin requires pRB and its family members, p107 and p130, for the stabilization of H4K20me3 by SUV4-20Hs, in a process that is independent of the E2F family [70]. Disruption of this heterochromatin structure due to pRB loss can result in centromere fusions, chromosome mis-segregation, and genomic instability [72].

The pRB/E2F complex can recruit enhancer of zeste homolog 2 (EZH2) to mediate H3K27me3 deposition to repress transcription during differentiation and stress [73]. H3K27me3 is also an important histone mark that represses heterochromatin at repetitive genomic regions, including tandem sequence repeats and interspersed repeats [74]. In this role, pRB, E2F1, and EZH2 can form a complex that directs H3K27me3 deposition to silence genomic repeat elements [75]. This cell-cycle-independent interaction between pRB and E2F1 initially recruits EZH2 to diverse repeat sequences, but pRB presence does not appear to be necessary for subsequent spreading of EZH2 and H3K27me3 [75]. Disrupting the ability of pRB to recruit EZH2 in mouse models results in cancer susceptibility, suggesting a role for pRB-EZH2 in tumor suppression [75].

The role of pRB in regulating histone methylation also extends to interactions with KDMs. pRB can bind to KDM1A (also known as LSD1) and KDM5A (also known as RBP2 or JARID1A), two KDMs that catalyze the removal of methyl groups from histone H3 lysine 4 (H3K4) [68,76]. Methylated H3K4 is a histone mark commonly found at actively transcribed promoter regions. KDM5A can demethylate H3K4me2/3, while KDM1A can demethylate H3K4me1/2. pRB binding to KMD1A represses transcription by recruiting it to demethylate H3K4 to pRB-regulated genes [68]. On the other hand, pRB binding to KDM5A displaces KDM5A and inhibits H3K4 demethylation, activating—rather than repressing—transcription [76]. Interestingly, the capacity of pRB to induce differentiation correlates with its ability to activate transcription together with transcription factors such as MYOD, CBFA1, and GRα [4,76]. KDM5A release from genes encoding mitochondrial proteins occurs early during differentiation and is required for the activation of differentiation markers. pRB can directly bind to and activate these KDM5A targets with mitochondrial functions potentially by sequestering KDM5A away from these genes [77]. Thus, pRB loss may also contribute to tumorigenesis through defects in cellular metabolism and terminal differentiation associated with its role in histone methylation.

## 6. pRB in DNA Methylation

DNA methylation involves the addition of a methyl group to the 5-carbon position of cytosine, forming 5-methylcytosine, at CpG dinucleotides [78,79]. Methylation of DNA is generally associated with an inactive chromatin through the inhibition of transcription factor binding [80], as well as allowing the occupation of methyl-CpG-binding proteins that facilitate transcriptional repression [81]. DNA methylation is catalyzed by enzymes in the DNA methyltransferase family (DNMTs), including DNMT1, which is associated with the maintenance of DNA methylation [82,83], and DNMT3A and DNMT3B, which are responsible for *de novo* DNA methylation [84]. pRB not only transcriptionally represses the expression of *DNMT1* and *DNMT3A*/*B* [85,86,87], but also physically interacts with DNMT1 and chromatin remodelers that can recruit DNMT1 and DNMT3A/B to affect DNA methylation [88,89,90,91].

As was discussed previously for histone modifiers, pRB transcriptional repression of E2F-regulated genes can also occur through direct recruitment of DNMT1. pRB can form a stable complex with E2F1, HDAC1, and DNMT1 and repress E2F-driven transcription [92]. Even though it is not clear whether the catalytic activity of DNMT1 is necessary for the E2F transcriptional repression of this complex, DNA methylation at E2F-dependent promoters is known to prevent the binding of E2F proteins [93]. The DNMT1/pRB interaction could serve as a way to enhance pRB-mediated repression of cell cycle progression during terminal differentiation as a mechanism to ensure genes involved in cell division are permanently turned off. Thus, it is likely that loss of functional pRB may contribute to aberrant DNMT1 localization and the resulting changes in global methylation observed in many cancer cells, including retinoblastoma.

The areas of the genome to be silenced by DNA methylation are recognized by chromatin-remodeling complexes that recruit DNMTs to the site. In this context, pRB is known to regulate the expression of chromatin remodeling proteins UHRF1 (ubiquitin-like, containing PHD and RING finger domains 1) and HELLS (helicase, lymphoid specific), which recruit DNMT1 and DNMT3, respectively. UHRF1 is a direct downstream target of the pRB/E2F pathway [88,89]. As a chromatin remodeler, UHRF1 recruits HDACs, HMTs, and DNMT1 to maintain heterochromatin during cell division (reviewed in [94]). pRB has an interesting connection with UHRF1 that goes beyond its transcriptional regulation as UHRF1 contains two LxCxE binding sequences that allow it to interact with pRB at the protein level, and UHRF1 can repress pRB gene transcription during S-phase entry [95]. UHRF1 overexpression is observed in multiple types of cancer with pRB loss, including retinoblastoma [96], with loss of UHRF1 capable of reversing the adverse effects associated with pRB loss [88]. Unpublished studies from our lab using genetic engineered mouse models of retinoblastoma also indicate that UHRF1 overexpression is required for the epigenetic changes that drive retinoblastoma tumor progression and in the absence of UHRF1, retinoblastoma tumors fail to form. HELLS is also transcriptionally regulated by the pRB/E2F pathway and overexpressed in multiple types of cancer, including retinoblastoma [90,96,97]. HELLS remodels chromatin to render DNA accessible to DNMT3 to support *de novo* DNA methylation and stable gene silencing during cellular differentiation. In the HELLS-mediated transcriptional repression, HELLS also acts as a recruiting factor for DNMT1 and HDACs to establish transcriptionally repressive chromatin [98]. In retinoblastoma, HELLS overexpression is critical for ectopic proliferation and tumor progression [91]. Consequently, inactivation of the pRB pathway alters DNMTs activity, leading to aberrant genomic DNA methylation patterns and malignant progression.

## 7. pRB Regulation of Non-Coding RNAs

The majority of transcriptional outcomes depend on non-coding RNAs (ncRNAs), including microRNAs (miRNAs) and long non-coding RNAs (lncRNAs). MiRNAs are short nucleotide sequences (~22 nucleotides) known to regulate gene expression post-transcriptionally. MiRNAs are transcribed by RNA polymerase II, cleaved in the nucleus by the endonuclease, Droscha, and then exported to the cytoplasm for further processing by another endonuclease, Dicer, allowing the transcript to become a mature miRNA duplex (reviewed in [99]). The miRNA can base pair to its corresponding regulatory site, forming the RNA-inducing silencing complex (RISC). LncRNAs are longer nucleotide sequences (>200 bp) that regulate gene expression at the transcriptional and post-transcriptional level. Primary lncRNAs are transcribed and transported into the nucleus similarly to miRNA transcripts but display similar properties of gene structure to protein-coding genes [100]. LncRNAs act as chromatin regulators by recruiting and interacting with chromatin remodeling enzymes, and as transcriptional regulators by binding to RNA-binding factors that can promote or repress gene transcription [101]. The transcriptional, post-transcriptional and epigenetic regulatory functions of ncRNAs can either be oncogenic or tumor suppressive. Here, we present pRB regulation of ncRNA, particularly during cancer.

Loss of pRB function and derepression of E2F-target genes results in deregulation of several ncRNA in RB (reviewed in [102]). However, whether or not several of these RNA molecules are directly regulated by pRB has not been thoroughly examined. One of the strongest evidence that pRB regulation of ncRNAs can have critical effects in tumorigenesis upon loss of pRB is portrayed by the miR-17~92 cluster [103]. In mouse models of retinoblastoma, loss of miR-17~92 completely suppresses retinoblastoma tumor formation [104], strongly supporting that miR-17~92 is regulated by pRB and critical for tumor development. In the absence of pRB, the cyclin-dependent kinase inhibitor p21^Cip1^ is upregulated; however, the overexpression of miR-17~92 caused by pRB-deficiency counteracts the upregulation of p21^Cip1^, leading to tumor formation [105]. Several other miRNAs that are transcriptionally controlled by activating E2Fs (E2F1, E2F2, and E2F3) during G1 are generally accepted to be transcriptionally repressed by pRB, as part of cell-cycle regulation by the pRB/E2F pathway, including miR-106b~25, miR-15b~16-2 and the let-7 family [106].

Several ncRNAs participate in auto-regulatory feedback loops. Therefore, many ncRNAs that regulate pRB may also be regulated by the pRB/E2F pathway. Research revealing ncRNA regulation by pRB should be further investigated for future engineering of potential therapeutic targets. In addition to the uncertainty regarding pRB regulation of ncRNAs, the degree of how much the regulatory networks differ between cancers compared to those in retinoblastoma is still unknown. A ncRNA biomarker for a specific cancer type may be different than those for retinoblastoma, even if that ncRNA is regulated by the pRB-E2F pathway. Further investigations on tissue specific expression patterns should be performed to reveal tissue-specific mechanisms of pRB regulation of ncRNAs.

## 8. Conclusions

Even though pRB and the pRB/E2F pathway have been extensively studied for decades, the molecular mechanisms behind the wide-ranging roles of this protein and pathway are still yet to be fully elucidated. As reviewed here, several of pRB functions, both within its canonical role in cell cycle control and other non-canonical cellular functions, are exerted through its interactions with epigenetic modifiers (summarized in Table 1). The intricacy of these multiprotein complexes and the cellular context under which they interact with pRB depict the diversity of pRB cellular roles and may explain why pRB inactivation is sufficient to cause cancer in some tissues (e.g., retinoblastoma) but not in others. Thus, understanding the context of these pRB interactions with multi-protein complexes that control nucleosome and chromatin modifications has the potential of providing alternative strategies for cancer therapy in a pathway that, at least to this day, remains largely undruggable.

As almost all human cancers carry abnormalities in the pRB/E2F pathway components including *INK4A*, *CCND*, *CDK4/6*, *RB1*, or *E2Fs*, genetic of functional inactivation of the pRB/E2F pathway seems to be indispensable for deregulated proliferation in most cancer settings [107]. Mutations in the *RB1* gene are found in retinoblastoma, osteosarcoma, and small cell lung cancer at a high frequency, and with less frequency in other types of cancer [6,107,108]. Mutations in upstream components such as *INK4A* are frequently detected in pancreatic cancer and non-small cell lung cancer, and *CCND* amplification is often found in breast cancer [6,107,109]. While these differences may be related to different tissue-specific mutational mechanisms, these observations also suggest that the pRB/E2F pathway is not strictly linear, and that loss of function due to genetic ablation of the *RB1* gene is not completely synonymous to loss of E2F binding activity due to hyper-phosphorylation of pRB. From the multiple examples presented, it is evident that many of the pRB functions are likely preserved when pRB is hyper-phosphorylated. Thus, not every one of pRB’s function should be considered as integral for its role as a tumor suppressor. Recent studies on the interplay between pRB and chromatin remodelers have been able to explain, in part, the role of pRB in the epigenetic mechanisms that lead to tumorigenesis. Whether these can be harnessed to develop novel cancer therapeutics remains to be determined.

## Figures and Tables

**Table 1:** Epigenetic regulators regulated by the pRB/E2F pathway

Epigenetic Regulation Type	Component	Relation with pRB/E2F Pathway	References
Nucleosome Mobilization			
	BRG1	E2F target and binds to pRB	[31]
	BRM	Binds to pRB	[34]
Histone Acetylation			
	HDAC1–3	Binds to pRB	[54,55]
	SIN3	Binds to pRB	[61,62]
	CTBP/CTIP	Binds to pRB	[61,62]
	CBP/p300	Binds to pRB	[58]
Histone Methylation			
	SUV39H1	Binds to pRB	[64,69]
	SUV4-20H1	Binds to pRB	[70]
	SUV4-20H2	Binds to pRB	[70]
	HP1	Binds to pRB	[64]
	EZH2	Binds to pRB	[73,75]
	KMD1A	Binds to pRB	[68]
	KMD5A	Binds to pRB	[76]
DNA Methylation			
	DNMT1	E2F target and binds to pRB.	[86]
	DNMT3	E2F target and binds to pRB.	[85]
	UHRF1	E2F target and binds to pRB.	[88,89,95]
	HELLS	E2F target and binds to pRB.	[90,96,97]
Non-coding RNAs			
	miR-17–92	E2F target	[103,104,105]
	miR-106b–25	E2F target	[106]
	miR-15b–16-2	E2F target	[106]
	let-7 family	E2F target	[106]

## References

[1] Harbour J.W., Dean D.C. (2000). The Rb/E2F pathway: Expanding roles and emerging paradigms. Genes Dev..

[2] Lee C., Chang J.H., Lee H.S., Cho Y. (2002). Structural basis for the recognition of the E2F transactivation domain by the retinoblastoma tumor suppressor. Genes Dev..

[3] Ianari A., Natale T., Calo E., Ferretti E., Alesse E., Screpanti I., Haigis K., Gulino A., Lees J.A. (2009). Proapoptotic function of the retinoblastoma tumor suppressor protein. Cancer Cell.

[4] Thomas D.M., Carty S.A., Piscopo D.M., Lee J.S., Wang W.F., Forrester W.C., Hinds P.W. (2001). The retinoblastoma protein acts as a transcriptional coactivator required for osteogenic differentiation. Mol. Cell.

[5] Narasimha A.M., Kaulich M., Shapiro G.S., Choi Y.J., Sicinski P., Dowdy S.F. (2014). Cyclin D activates the Rb tumor suppressor by mono-phosphorylation. eLife.

[6] Dyson N.J. (2016). RB1: A prototype tumor suppressor and an enigma. Genes Dev..

[7] Magnaghi-Jaulin L., Groisman R., Naguibneva I., Robin P., Lorain S., Le Villain J.P., Troalen F., Trouche D., Harel-Bellan A. (1998). Retinoblastoma protein represses transcription by recruiting a histone deacetylase. Nature.

[8] Sanidas I., Morris R., Fella K.A., Rumde P.H., Boukhali M., Tai E.C., Ting D.T., Lawrence M.S., Haas W., Dyson N.J. (2019). A Code of Mono-phosphorylation Modulates the Function of RB. Mol. Cell.

[9] Zhang J., Benavente C.A., McEvoy J., Flores-Otero J., Ding L., Chen X., Ulyanov A., Wu G., Wilson M., Wang J. (2012). A novel retinoblastoma therapy from genomic and epigenetic analyses. Nature.

[10] Wang J.Y., Knudsen E.S., Welch P.J. (1994). The retinoblastoma tumor suppressor protein. Adv. Cancer Res..

[11] Welch P.J., Wang J.Y. (1995). Disruption of retinoblastoma protein function by coexpression of its C pocket fragment. Genes Dev..

[12] Dick F.A., Rubin S.M. (2013). Molecular mechanisms underlying RB protein function. Nat. Rev. Mol. Cell Biol..

[13] Macdonald J.I., Dick F.A. (2012). Posttranslational modifications of the retinoblastoma tumor suppressor protein as determinants of function. Genes Cancer.

[14] Hassler M., Singh S., Yue W.W., Luczynski M., Lakbir R., Sanchez-Sanchez F., Bader T., Pearl L.H., Mittnacht S. (2007). Crystal structure of the retinoblastoma protein N domain provides insight into tumor suppression, ligand interaction, and holoprotein architecture. Mol. Cell.

[15] Rubin S.M., Gall A.L., Zheng N., Pavletich N.P. (2005). Structure of the Rb C-terminal domain bound to E2F1-DP1: A mechanism for phosphorylation-induced E2F release. Cell.

[16] Burke J.R., Deshong A.J., Pelton J.G., Rubin S.M. (2010). Phosphorylation-induced conformational changes in the retinoblastoma protein inhibit E2F transactivation domain binding. J. Biol. Chem..

[17] Burke J.R., Hura G.L., Rubin S.M. (2012). Structures of inactive retinoblastoma protein reveal multiple mechanisms for cell cycle control. Genes Dev..

[18] Weinberg R.A. (1995). The retinoblastoma protein and cell cycle control. Cell.

[19] Phelan M.L., Sif S., Narlikar G.J., Kingston R.E. (1999). Reconstitution of a core chromatin remodeling complex from SWI/SNF subunits. Mol. Cell.

[20] Muchardt C., Yaniv M. (1999). ATP-dependent chromatin remodelling: SWI/SNF and Co. are on the job. J. Mol. Biol..

[21] Tyler J.K., Kadonaga J.T. (1999). The "dark side" of chromatin remodeling: Repressive effects on transcription. Cell.

[22] Laurent B.C., Treitel M.A., Carlson M. (1991). Functional interdependence of the yeast SNF2, SNF5, and SNF6 proteins in transcriptional activation. Proc. Natl. Acad. Sci. USA.

[23] Dingwall A.K., Beek S.J., McCallum C.M., Tamkun J.W., Kalpana G.V., Goff S.P., Scott M.P. (1995). The Drosophila snr1 and brm proteins are related to yeast SWI/SNF proteins and are components of a large protein complex. Mol. Biol. Cell.

[24] Papoulas O., Beek S.J., Moseley S.L., McCallum C.M., Sarte M., Shearn A., Tamkun J.W. (1998). The Drosophila trithorax group proteins BRM, ASH1 and ASH2 are subunits of distinct protein complexes. Development.

[25] Kwon H., Imbalzano A.N., Khavari P.A., Kingston R.E., Green M.R. (1994). Nucleosome disruption and enhancement of activator binding by a human SW1/SNF complex. Nature.

[26] Wang W., Xue Y., Zhou S., Kuo A., Cairns B.R., Crabtree G.R. (1996). Diversity and specialization of mammalian SWI/SNF complexes. Genes Dev..

[27] Sif S., Stukenberg P.T., Kirschner M.W., Kingston R.E. (1998). Mitotic inactivation of a human SWI/SNF chromatin remodeling complex. Genes Dev..

[28] Hamiche A., Sandaltzopoulos R., Gdula D.A., Wu C. (1999). ATP-dependent histone octamer sliding mediated by the chromatin remodeling complex NURF. Cell.

[29] Khavari P.A., Peterson C.L., Tamkun J.W., Mendel D.B., Crabtree G.R. (1993). BRG1 contains a conserved domain of the SWI2/SNF2 family necessary for normal mitotic growth and transcription. Nature.

[30] Muchardt C., Yaniv M. (1993). A human homologue of Saccharomyces cerevisiae SNF2/SWI2 and Drosophila brm genes potentiates transcriptional activation by the glucocorticoid receptor. EMBO J..

[31] Dunaief J.L., Strober B.E., Guha S., Khavari P.A., Alin K., Luban J., Begemann M., Crabtree G.R., Goff S.P. (1994). The retinoblastoma protein and BRG1 form a complex and cooperate to induce cell cycle arrest. Cell.

[32] Singh P., Coe J., Hong W. (1995). A role for retinoblastoma protein in potentiating transcriptional activation by the glucocorticoid receptor. Nature.

[33] Strober B.E., Dunaief J.L., Guha, Goff S.P. (1996). Functional interactions between the hBRM/hBRG1 transcriptional activators and the pRB family of proteins. Mol. Cell. Biol..

[34] Trouche D., Le Chalony C., Muchardt C., Yaniv M., Kouzarides T. (1997). RB and hbrm cooperate to repress the activation functions of E2F1. Proc. Natl. Acad. Sci. USA.

[35] Zhu L., van den Heuvel S., Helin K., Fattaey A., Ewen M., Livingston D., Dyson N., Harlow E. (1993). Inhibition of cell proliferation by p107, a relative of the retinoblastoma protein. Genes Dev..

[36] Strobeck M.W., Knudsen K.E., Fribourg A.F., DeCristofaro M.F., Weissman B.E., Imbalzano A.N., Knudsen E.S. (2000). BRG-1 is required for RB-mediated cell cycle arrest. Proc. Natl. Acad. Sci. USA.

[37] Reisman D.N., Strobeck M.W., Betz B.L., Sciariotta J., Funkhouser W., Murchardt C., Yaniv M., Sherman L.S., Knudsen E.S., Weissman B.E. (2002). Concomitant down-regulation of BRM and BRG1 in human tumor cell lines: Differential effects on RB-mediated growth arrest vs CD44 expression. Oncogene.

[38] Bartlett C., Orvis T.J., Rosson G.S., Weissman B.E. (2011). BRG1 mutations found in human cancer cell lines inactivate Rb-mediated cell-cycle arrest. J. Cell. Physiol..

[39] Strobeck M.W., Reisman D.N., Gunawardena R.W., Betz B.L., Angus S.P., Knudsen K.E., Kowalik T.F., Weissman B.E., Knudsen E.S. (2002). Compensation of BRG-1 function by Brm: Insight into the role of the core SWI-SNF subunits in retinoblastoma tumor suppressor signaling. J. Biol. Chem..

[40] Kang H., Cui K., Zhao K. (2004). BRG1 controls the activity of the retinoblastoma protein via regulation of p21CIP1/WAF1/SDI. Mol. Cell. Biol..

[41] Zhang H.S., Gavin M., Dahiya A., Postigo A.A., Ma D., Luo R.X., Harbour J.W., Dean D.C. (2000). Exit from G1 and S phase of the cell cycle is regulated by repressor complexes containing HDAC-Rb-hSWI/SNF and Rb-hSWI/SNF. Cell.

[42] Muchardt C., Reyes J.C., Bourachot B., Leguoy E., Yaniv M. (1996). The hbrm and BRG-1 proteins, components of the human SNF/SWI complex, are phosphorylated and excluded from the condensed chromosomes during mitosis. EMBO J..

[43] Shanahan F., Seghezzi W., Parry D., Mahony D., Lees E. (1999). Cyclin E associates with BAF155 and BRG1, components of the mammalian SWI-SNF complex, and alters the ability of BRG1 to induce growth arrest. Mol. Cell. Biol..

[44] Velez-Cruz R., Manickavinayaham S., Biswas A.K., Clary R.W., Premkumar T., Cole F., Johnson D.G. (2016). RB localizes to DNA double-strand breaks and promotes DNA end resection and homologous recombination through the recruitment of BRG1. Genes Dev..

[45] Campanero M.R., Flemington E.K. (1997). Regulation of E2F through ubiquitin-proteasome-dependent degradation: Stabilization by the pRB tumor suppressor protein. Proc. Natl. Acad. Sci. USA.

[46] Kadoch C., Hargreaves D.C., Hodges C., Elias L., Ho L., Ranish J., Crabtree G.R. (2013). Proteomic and bioinformatic analysis of mammalian SWI/SNF complexes identifies extensive roles in human malignancy. Nat. Genet..

[47] Wong A.K., Shanahan F., Chen Y., Lian L., Ha P., Hendricks K., Ghaffari S., Iliev D., Penn B., Woodland A.M. (2000). BRG1, a component of the SWI-SNF complex, is mutated in multiple human tumor cell lines. Cancer Res..

[48] Reisman D.N., Sciarrotta J., Wang W., Funkhouser W.K., Weissman B.E. (2003). Loss of BRG1/BRM in human lung cancer cell lines and primary lung cancers: Correlation with poor prognosis. Cancer Res..

[49] Jubierre L., Soriano A., Planells-Ferrer L., Paris-Coderch L., Tenbaum S.P., Romero O.A., Moubarak R.S., Almazan-Moga A., Molist C., Roma J. (2016). BRG1/SMARCA4 is essential for neuroblastoma cell viability through modulation of cell death and survival pathways. Oncogene.

[50] Bai J., Mei P., Zhang C., Chen F., Li C., Pan Z., Liu H., Zheng J. (2013). BRG1 is a prognostic marker and potential therapeutic target in human breast cancer. PLoS ONE.

[51] Muthuswami R., Bailey L., Rakesh R., Imbalzano A.N., Nickerson J.A., Hockensmith J.W. (2019). BRG1 is a prognostic indicator and a potential therapeutic target for prostate cancer. J. Cell. Physiol..

[52] Aldiri I., Ajioka I., Xu B., Zhang J., Chen X., Benavente C., Finkelstein D., Johnson D., Akiyama J., Pennacchio L.A. (2015). Brg1 coordinates multiple processes during retinogenesis and is a tumor suppressor in retinoblastoma. Development.

[53] Roth S.Y., Denu J.M., Allis C.D. (2001). Histone acetyltransferases. Annu. Rev. Biochem..

[54] Brehm A., Miska E.A., McCance D.J., Reid J.L., Bannister A.J., Kouzarides T. (1998). Retinoblastoma protein recruits histone deacetylase to repress transcription. Nature.

[55] Puri P.L., Iezzi S., Stiegler P., Chen T.T., Schiltz R.L., Muscat G.E., Giordano A., Kedes L., Wang J.Y., Sartorelli V. (2001). Class I histone deacetylases sequentially interact with MyoD and pRb during skeletal myogenesis. Mol. Cell.

[56] MacLellan W.R., Xiao G., Abdellatif M., Schneider M.D. (2000). A novel Rb- and p300-binding protein inhibits transactivation by MyoD. Mol. Cell. Biol..

[57] Siegert J.L., Robbins P.D. (1999). Rb inhibits the intrinsic kinase activity of TATA-binding protein-associated factor TAFII250. Mol. Cell. Biol..

[58] Manickavinayaham S., Velez-Cruz R., Biswas A.K., Bedford E., Klein B.J., Kutateladze T.G., Liu B., Bedford M.T., Johnson D.G. (2019). E2F1 acetylation directs p300/CBP-mediated histone acetylation at DNA double-strand breaks to facilitate repair. Nat. Commun..

[59] Dahiya A., Gavin M.R., Luo R.X., Dean D.C. (2000). Role of the LXCXE binding site in Rb function. Mol. Cell. Biol..

[60] Lai A., Lee J.M., Yang W.M., DeCaprio J.A., Kaelin W.G., Seto E., Branton P.E. (1999). RBP1 recruits both histone deacetylase-dependent and -independent repression activities to retinoblastoma family proteins. Mol. Cell. Biol..

[61] Lai A., Kennedy B.K., Barbie D.A., Bertos N.R., Yang X.J., Theberge M.C., Tsai S.C., Seto E., Zhang Y., Kuzmichev A. (2001). RBP1 recruits the mSIN3-histone deacetylase complex to the pocket of retinoblastoma tumor suppressor family proteins found in limited discrete regions of the nucleus at growth arrest. Mol. Cell. Biol..

[62] Meloni A.R., Smith E.J., Nevins J.R. (1999). A mechanism for Rb/p130-mediated transcription repression involving recruitment of the CtBP corepressor. Proc. Natl. Acad. Sci. USA.

[63] Morrison A.J., Sardet C., Herrera R.E. (2002). Retinoblastoma protein transcriptional repression through histone deacetylation of a single nucleosome. Mol. Cell. Biol..

[64] Nielsen S.J., Schneider R., Bauer U.M., Bannister A.J., Morrison A., O’Carroll D., Firestein R., Cleary M., Jenuwein T., Herrera R.E. (2001). Rb targets histone H3 methylation and HP1 to promoters. Nature.

[65] Vaute O., Nicolas E., Vandel L., Trouche D. (2002). Functional and physical interaction between the histone methyl transferase Suv39H1 and histone deacetylases. Nucleic Acids Res..

[66] Montoya-Durango D.E., Liu Y., Teneng I., Kalbfleisch T., Lacy M.E., Steffen M.C., Ramos K.S. (2009). Epigenetic control of mammalian LINE-1 retrotransposon by retinoblastoma proteins. Mutat. Res..

[67] Cook R., Zoumpoulidou G., Luczynski M.T., Rieger S., Moquet J., Spanswick V.J., Hartley J.A., Rothkamm K., Huang P.H., Mittnacht S. (2015). Direct involvement of retinoblastoma family proteins in DNA repair by non-homologous end-joining. Cell Rep..

[68] Chau C.M., Deng Z., Kang H., Lieberman P.M. (2008). Cell cycle association of the retinoblastoma protein Rb and the histone demethylase LSD1 with the Epstein-Barr virus latency promoter Cp. J. Virol..

[69] Vandel L., Nicolas E., Vaute O., Ferreira R., Ait-Si-Ali S., Trouche D. (2001). Transcriptional repression by the retinoblastoma protein through the recruitment of a histone methyltransferase. Mol. Cell. Biol..

[70] Gonzalo S., Garcia-Cao M., Fraga M.F., Schotta G., Peters A.H., Cotter S.E., Eguia R., Dean D.C., Esteller M., Jenuwein T. (2005). Role of the RB1 family in stabilizing histone methylation at constitutive heterochromatin. Nat. Cell Biol..

[71] Narita M., Nunez S., Heard E., Narita M., Lin A.W., Hearn S.A., Spector D.L., Hannon G.J., Lowe S.W. (2003). Rb-mediated heterochromatin formation and silencing of E2F target genes during cellular senescence. Cell.

[72] Isaac C.E., Francis S.M., Martens A.L., Julian L.M., Seifried L.A., Erdmann N., Binne U.K., Harrington L., Sicinski P., Berube N.G. (2006). The retinoblastoma protein regulates pericentric heterochromatin. Mol. Cell. Biol..

[73] Blais A., van Oevelen C.J., Margueron R., Acosta-Alvear D., Dynlacht B.D. (2007). Retinoblastoma tumor suppressor protein-dependent methylation of histone H3 lysine 27 is associated with irreversible cell cycle exit. J. Cell Biol..

[74] Day D.S., Luquette L.J., Park P.J., Kharchenko P.V. (2010). Estimating enrichment of repetitive elements from high-throughput sequence data. Genome Biol..

[75] Ishak C.A., Marshall A.E., Passos D.T., White C.R., Kim S.J., Cecchini M.J., Ferwati S., MacDonald W.A., Howlett C.J., Welch I.D. (2016). An RB-EZH2 Complex Mediates Silencing of Repetitive DNA Sequences. Mol. Cell.

[76] Benevolenskaya E.V., Murray H.L., Branton P., Young R.A., Kaelin W.G. (2005). Binding of pRB to the PHD protein RBP2 promotes cellular differentiation. Mol. Cell.

[77] Varaljai R., Islam A.B., Beshiri M.L., Rehman J., Lopez-Bigas N., Benevolenskaya E.V. (2015). Increased mitochondrial function downstream from KDM5A histone demethylase rescues differentiation in pRB-deficient cells. Genes Dev..

[78] Gardiner-Garden M., Frommer M. (1987). CpG islands in vertebrate genomes. J. Mol. Biol..

[79] Hotchkiss R.D. (1948). The quantitative separation of purines, pyrimidines, and nucleosides by paper chromatography. J. Biol. Chem..

[80] Dantas Machado A.C., Zhou T., Rao S., Goel P., Rastogi C., Lazarovici A., Bussemaker H.J., Rohs R. (2015). Evolving insights on how cytosine methylation affects protein-DNA binding. Brief. Funct. Genom..

[81] Ballestar E., Wolffe A.P. (2001). Methyl-CpG-binding proteins. Targeting specific gene repression. Eur. J. Biochem..

[82] Hermann A., Goyal R., Jeltsch A. (2004). The Dnmt1 DNA-(cytosine-C5)-methyltransferase methylates DNA processively with high preference for hemimethylated target sites. J. Biol. Chem..

[83] Mortusewicz O., Schermelleh L., Walter J., Cardoso M.C., Leonhardt H. (2005). Recruitment of DNA methyltransferase I to DNA repair sites. Proc. Natl. Acad. Sci. USA.

[84] Okano M., Bell D.W., Haber D.A., Li E. (1999). DNA methyltransferases Dnmt3a and Dnmt3b are essential for de novo methylation and mammalian development. Cell.

[85] Tang Y.A., Lin R.K., Tsai Y.T., Hsu H.S., Yang Y.C., Chen C.Y., Wang Y.C. (2012). MDM2 overexpression deregulates the transcriptional control of RB/E2F leading to DNA methyltransferase 3A overexpression in lung cancer. Clin. Cancer Res..

[86] McCabe M.T., Davis J.N., Day M.L. (2005). Regulation of DNA methyltransferase 1 by the pRb/E2F1 pathway. Cancer Res..

[87] Tang Y.A., Tsai Y.T., Lin R.K., Hsu H.S., Chen C.Y., Wang Y.C. (2014). Deregulation of p53 and RB Transcriptional Control of DNA Methyltransferases in Lung Cancer. J. Cancer Res. Pract..

[88] Wu S.C., Lopez J., Salcido K., Zocchi L., Wu J., Benavente C.A. (2020). UHRF1 drives the poor prognosis associated with RB loss in osteosarcoma. bioRxiv.

[89] Magri L., Swiss V.A., Jablonska B., Lei L., Pedre X., Walsh M., Zhang W., Gallo V., Canoll P., Casaccia P. (2014). E2F1 coregulates cell cycle genes and chromatin components during the transition of oligodendrocyte progenitors from proliferation to differentiation. J. Neurosci..

[90] Wu S.C., Benavente C.A. (2018). Chromatin remodeling protein HELLS is upregulated by inactivation of the RB-E2F pathway and is nonessential for osteosarcoma tumorigenesis. Oncotarget.

[91] Zocchi L., Mehta A., Wu S.C., Wu J., Gu Y., Wang J., Suh S., Spitale R.C., Benavente C.A. (2020). Chromatin remodeling protein HELLS is critical for retinoblastoma tumor initiation and progression. Oncogenesis.

[92] Robertson K.D., Ait-Si-Ali S., Yokochi T., Wade P.A., Jones P.L., Wolffe A.P. (2000). DNMT1 forms a complex with Rb, E2F1 and HDAC1 and represses transcription from E2F-responsive promoters. Nat. Genet..

[93] Campanero M.R., Armstrong M.I., Flemington E.K. (2000). CpG methylation as a mechanism for the regulation of E2F activity. Proc. Natl. Acad. Sci. USA.

[94] Bronner C., Alhosin M., Hamiche A., Mousli M. (2019). Coordinated Dialogue between UHRF1 and DNMT1 to Ensure Faithful Inheritance of Methylated DNA Patterns. Genes.

[95] Jeanblanc M., Mousli M., Hopfner R., Bathami K., Martinet N., Abbady A.Q., Siffert J.C., Mathieu E., Muller C.D., Bronner C. (2005). The retinoblastoma gene and its product are targeted by ICBP90: A key mechanism in the G1/S transition during the cell cycle. Oncogene.

[96] Benavente C.A., Finkelstein D., Johnson D.A., Marine J.C., Ashery-Padan R., Dyer M.A. (2014). Chromatin remodelers HELLS and UHRF1 mediate the epigenetic deregulation of genes that drive retinoblastoma tumor progression. Oncotarget.

[97] Niu J., Chen T., Han L., Wang P., Li N., Tong T. (2011). Transcriptional activation of the senescence regulator Lsh by E2F1. Mech. Ageing Dev..

[98] Myant K., Stancheva I. (2008). LSH cooperates with DNA methyltransferases to repress transcription. Mol. Cell. Biol..

[99] Gebert L.F.R., MacRae I.J. (2019). Regulation of microRNA function in animals. Nat. Rev. Mol. Cell. Biol..

[100] Derrien T., Johnson R., Bussotti G., Tanzer A., Djebali S., Tilgner H., Guernec G., Martin D., Merkel A., Knowles D.G. (2012). The GENCODE v7 catalog of human long noncoding RNAs: Analysis of their gene structure, evolution, and expression. Genome Res..

[101] Guttman M., Amit I., Garber M., French C., Lin M.F., Feldser D., Huarte M., Zuk O., Carey B.W., Cassady J.P. (2009). Chromatin signature reveals over a thousand highly conserved large non-coding RNAs in mammals. Nature.

[102] Plousiou M., Vannini I. (2019). Non-Coding RNAs in Retinoblastoma. Front. Genet..

[103] Aguda B.D., Kim Y., Piper-Hunter M.G., Friedman A., Marsh C.B. (2008). MicroRNA regulation of a cancer network: Consequences of the feedback loops involving miR-17-92, E2F, and Myc. Proc. Natl. Acad. Sci. USA.

[104] Nittner D., Lambertz I., Clermont F., Mestdagh P., Kohler C., Nielsen S.J., Jochemsen A., Speleman F., Vandesompele J., Dyer M.A. (2012). Synthetic lethality between Rb, p53 and Dicer or miR-17-92 in retinal progenitors suppresses retinoblastoma formation. Nat. Cell Biol..

[105] Conkrite K., Sundby M., Mukai S., Thomson J.M., Mu D., Hammond S.M., MacPherson D. (2011). miR-17~92 cooperates with RB pathway mutations to promote retinoblastoma. Genes Dev..

[106] Bueno M.J., Gomez de Cedron M., Laresgoiti U., Fernandez-Piqueras J., Zubiaga A.M., Malumbres M. (2010). Multiple E2F-induced microRNAs prevent replicative stress in response to mitogenic signaling. Mol. Cell. Biol..

[107] Burkhart D.L., Sage J. (2008). Cellular mechanisms of tumour suppression by the retinoblastoma gene. Nat. Rev. Cancer.

[108] Friend S.H., Bernards R., Rogelj S., Weinberg R.A., Rapaport J.M., Albert D.M., Dryja T.P. (1986). A human DNA segment with properties of the gene that predisposes to retinoblastoma and osteosarcoma. Nature.

[109] Knudsen E.S., Knudsen K.E. (2008). Tailoring to RB: Tumour suppressor status and therapeutic response. Nat. Rev. Cancer.

